# Inhibitory Mechanism of Baicalein on Acetylcholinesterase: Inhibitory Interaction, Conformational Change, and Computational Simulation

**DOI:** 10.3390/foods11020168

**Published:** 2022-01-10

**Authors:** Yijing Liao, Xing Hu, Junhui Pan, Guowen Zhang

**Affiliations:** 1 State Key Laboratory of Food Science and Technology, Nanchang University, Nanchang 330047, China; yijingliao@ncu.edu.cn (Y.L.); hx0726@ncu.edu.cn (X.H.); panjunhui@ncu.edu.cn (J.P.); 2 School of Pharmacy, Nanchang University, Nanchang 330006, China

**Keywords:** baicalein, acetylcholinesterase, inhibitory mechanism, conformational change, molecular docking, molecular dynamics simulation

## Abstract

Alzheimer’s disease (AD) is the most prevalent chronic neurodegenerative disease in elderly individuals, causing dementia. Acetylcholinesterase (AChE) is regarded as one of the most popular drug targets for AD. Herbal secondary metabolites are frequently cited as a major source of AChE inhibitors. In the current study, baicalein, a typical bioactive flavonoid, was found to inhibit AChE competitively, with an associated IC_50_ value of 6.42 ± 0.07 µM, through a monophasic kinetic process. The AChE fluorescence quenching by baicalein was a static process. The binding constant between baicalein and AChE was an order of magnitude of 10^4^ L mol^−1^, and hydrogen bonding and hydrophobic interaction were the major forces for forming the baicalein−AChE complex. Circular dichroism analysis revealed that baicalein caused the AChE structure to shrink and increased its surface hydrophobicity by increasing the α-helix and β-turn contents and decreasing the β-sheet and random coil structure content. Molecular docking revealed that baicalein predominated at the active site of AChE, likely tightening the gorge entrance and preventing the substrate from entering and binding with the enzyme, resulting in AChE inhibition. The preceding findings were confirmed by molecular dynamics simulation. The current study provides an insight into the molecular-level mechanism of baicalein interaction with AChE, which may offer new ideas for the research and development of anti-AD functional foods and drugs.

## 1. Introduction

Alzheimer’s disease (AD), a progressive neurodegenerative disease featuring hidden onset, is clinically the most prevalent dementia form, which manifests as cognitive impairment, such as memory loss, out-of-control behaviour, and emotional disorder [1,2]. According to Alzheimer’s Disease International’s data in September 2021, the number of AD patients worldwide is 55 million. With the increase in the ageing population, the number of AD patients is estimated to reach 78 million by 2030 and 139 million by 2050, with a new AD case appearing every 3 s [3]. AD has become a major challenge impacting the health of older adults and has become a public health concern [4].

**Figure 1:**
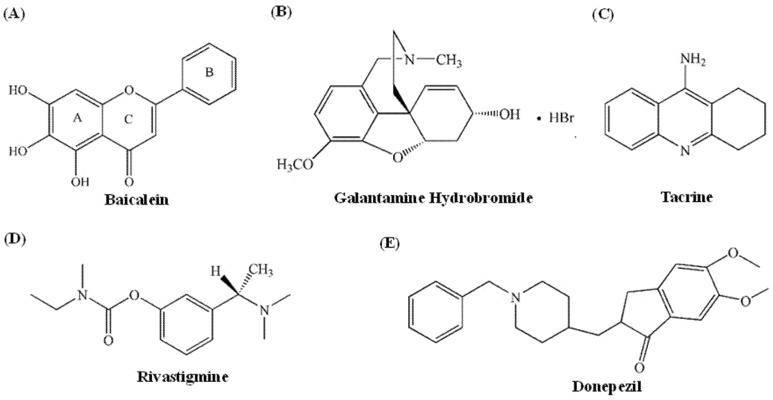
Structure of baicalein (**A**) and FDA-approved AChE inhibitors (**B**–**E**).

The aetiology of AD is currently unknown [5]. Scholars both at home and abroad have proposed multiple hypotheses about its pathogenesis, among which the “cholinergic hypothesis” is the most developed and well-known [6,7,8]. Degeneration of basal cholinergic neurons in the forebrain and loss of associated cholinergic neurotransmitters in the cerebral cortex and other regions lead to the degradation of cognitive function in AD patients [9]. Acetylcholinesterase (AChE, Acetylcholinesterase, E.C.3.1.1.7) is a serine protease that catalyses the hydrolysis of acetylcholine (ACh), an essential neurotransmitter in vivo, and decomposes it into acetic acid and choline, thus blocking nerve impulse transmission [10,11]. According to research, AD patients lack the cholinergic nerve cells in the basal forebrain and thus exhibit enhanced AChE activity and decreased acetylcholine content [12]. As a result, acetylcholinesterase inhibitors targeting AChE are mainly used to treat AD in the clinic, according to the “cholinergic hypothesis.” Four of the five anti-AD drugs approved by the FDA include AChE inhibitors (namely tacrine, donepezil, galanthamine, and rivastigmine, Figure 1) [13,14]. Tacrine, however, was withdrawn in 2013 owing to severe hepatotoxicity and other side effects [15]. The other three clinically utilised drugs have varying side effects and can only treat mild-to-moderate AD symptoms; therefore, in-depth research is required to achieve satisfactory therapeutic results [16]. The failure of clinical AD drug research teaches us that drug selection must be based on both safety and targeting, and screening AD inhibitors from natural products is an effective strategy [17].

Foods high in flavonoids have been found to reduce the neurodegenerative pathological process of AD animal models and improve cognitive function [18]. Epidemiological studies have also demonstrated that eating a substantial amount of foods rich in flavonoids can improve normal cognitive function and reduce the incidence of dementia in the local population [19]. Flavonoids are highly effective in alleviating reversible neurodegenerative pathological processes and improving age-related cognitive decline [20] and thus have become a hot spot in the research and development of anti-AD drugs and functional food factors both at home and abroad.

Baicalein (5,6,7-trihydroxyflavone, as shown in Figure 1A) is an effective active component monomer of flavonoids extracted from the root of *Scutellaria baicalensis* Georgi [21], and it exerts numerous pharmacological effects, including antioxidant [22], scavenging oxygen free radicals [23], anti-inflammatory [24], antimicrobial [25], anti-carcinogenic [26] and neuroprotective effects [27]. The effects of baicalein on alleviating AD and improving cognition and brain protection have been reported [28,29,30]. Baicalein can activate the PI3K pathway, inhibit the levels of GSK3β and BACE1, reduce the content of total Aβ, and play a neuroprotective role in mice, thereby enhancing memory [31]. Wei [32] confirmed that baicalein can effectively alleviate cognitive impairment induced by Aβ _1-40_ in rats and change the protein expression level in the cerebral cortex and hippocampus. Studies by Han [33] and other scholars have shown that baicalein is a selective and specific inhibitor of BACE and AChE that can be utilised to prevent and treat AD. Xie [34] investigated the inhibitory effect of 20 flavonoids on AChE and explored their structure−activity relationship in the preliminary stages. However, the detailed molecular mechanism of baicalein against AChE is yet to be explored.

In the current study, UV–Vis absorption, fluorescence, and circular dichroism (CD) spectroscopy were used to investigate the inhibitory activity, inhibitory kinetics, binding properties, number of binding sites, type of acting force, and effect on the conformation of AChE by baicalein. A PC12 cytotoxicity test was performed to assess the safety of baicalein. For the interaction of baicalein with AChE, the molecular simulation was utilised to predict binding sites, binding conditions, and major amino acid residues. Molecular dynamics (M.D.) simulation was used to investigate the stability of protein skeleton, peptide flexibility, and free energy of amino acid residues before and after the binding of baicalein with AChE. The findings may provide novel insights into the mechanism of baicalein in AChE inhibition and serve as a reference for the research and development of flavonoids, such as baicalein, as anti-AD food functional factors and drugs.

## 2. Materials and Methods

### 2.1. Materials

AChE (137 U/mg, Electrophorus electricus) was provided by Sigma-Aldrich Co. (St. Louis, MO, USA), and their stock solutions with 10 U/mL concentrations were prepared with 0.1 M phosphate-buffered saline (pH 7.6) and stored at −20 °C. Baicalein (purity ≥ 98%) was obtained from the National Institute for the Control of Pharmaceutical and Biological Products (Beijing, China) and dissolved in ethanol to prepare the stock solution (5 mM). 5,5-Dithiobis-(2-nitrobenzoic acid) (DTNB), acetylthiocholine iodide (ATCI), galathamine hydrobromide, and 3-(4, 5-dimethylthiazol-2-yl)-2,5-diphenyltetrazolium bromide (MTT) were provided by Aladdin Chemistry Co., Ltd. (Shanghai, China). Foetal bovine serum (FBS) was purchased from Gibco (Carlsbad, CA, USA). Dulbecco’s modified Eagle’s medium (DMEM), trypsin, and other cell culture reagents were purchased from Solarbio (Beijing, China). All remaining reagents were of analytical grade.

### 2.2. Enzyme Activity Assay

AChE activity was evaluated per Ellman’s method, albeit with a slight modification [35]. Quartz cuvettes (3 mL) were utilised for measuring the assay mixture activity. Solutions containing varying amounts of baicalein, phosphate-buffered saline (0.1 M, pH 7.6), and 50 µL of AChE (2 U/mL, 3.4 × 10^−8^ M) were cultivated in 30 min at 25 °C. After the pre-incubation period, 50 μL of 5, 5-dithiobis-(2-nitrobenzoic) acid (DNTB, 5 mM) was added. The reaction was then initiated with 50 μL of acetylthiocholine iodide (ATCI, 15 mM). The absorbance at 412 nm was monitored every 5 s with the TU−1901 dual-beam UV–Vis Spectrophotometer (Persee; Beijing, China). The samples were measured thrice, and the average value was considered. Enzyme activity without baicalein was identified to be 100%. Herein, the measured half-inhibitory concentration (IC_50_) for baicalein and the relative enzymatic activity agreed with those reported previously [36]. Galantamine hydrobromide served as the positive control.

### 2.3. Kinetic Analysis of the Inhibitory Type

To assess the kinetic mode of AChE inhibition displayed by baicalein, the inhibitory effect was determined with four different concentrations of baicalein (0, 4, 8, and 10 µM) when different substrate concentrations of ATCI coexisted in the same way of as the AChE activity assay. The Lineweaver–Burk plots and Michaelis–Menten enzyme kinetics were applied to analyse the inhibitory type. For competitive type inhibition, the dynamic double reciprocal equation was used as follows [37]:

(1) $$\frac{1}{v}=\frac{K_{m}}{v_{\max}}\left(1+\frac{\left(I\right)\;}{K_{i}}\right)\frac{1}{\left(S\right)}+\frac{1}{v_{\max}}$$

(2) $$K_{m}^{\mathrm{app}}=\frac{K_{m}\left(I\right)}{K_{i}}+K_{m}$$

where (*I*) and (*S*) denote the inhibitor and substrate concentrations, respectively; ν represents the enzyme reaction rate irrespective of the presence of baicalein; ν_max_ is the maximum catalytic reaction rate; *K_i_*, *K_m_*, and $K_{m}^{\mathrm{app}}$ stand for the inhibition constant, Michaelis−Menten constant, and apparent Michaelis constant, respectively.

The kinetic process and rate constants for enzyme inactivation were determined through the time-course test. Baicalein with concentrations of 2, 4, 8, and 16 µM was used for analysing the time-course. Enzyme activity at each concentration was measured every 3 min for 0–30 min, every 6 min for 30–60 min, and then at 70 and 80 min.

### 2.4. Fluorescence Spectrum Measurement

A spectrofluorometer (model F-7000; Hitachi, Tokyo, Japan) containing a thermostat bath was used to measure the fluorescence spectra. The AChE solution (2.5 mL; 0.17 µM) was introduced into a 1.0 cm path length quartz cell, followed by titration through the successive addition of 2.0 mM of diluted baicalein solution (to obtain the concentration range of 0–16 µM). The well-mixed solutions were set aside for three min for equilibration purposes. The fluorescence spectra were measured in the 290–450 nm range, and the relevant excitation wavelength set was 280 nm at 25 °C, 31°C, and 37 °C. Excitation and emission bandwidths were 2.5 nm. In addition, synchronous fluorescence spectra for AChE with baicalein were conducted by fixing the interval of excitation and emission wavelength constants at 15 nm and 60 nm. Fluorescence data were amended according to our previous reports to erase the re-absorption possibility and the inner filter effects of UV absorption [38].

### 2.5. CD Spectra Measurements

Far-UV CD spectra were measured using a spectrometer (MOS 450 CD; Bio-Logic, Claix, France) with a 1.0 mm path length quartz cuvette. All the spectra were measured in the 200–240 nm range at 25 °C, and the associated scan speed was 60 nm min^−1^. The AChE in the PBS was completely blended with baicalein at varying concentrations and then equilibrated for three min before CD measurements. The spectra were amended by subtracting the spectrum of the buffer with that of baicalein at the same concentration as the sample solution. Three scans were conducted, and the ellipticity in millidegrees indicated the results. The contents of the discrepant secondary structures (α-helix, β-sheet, β-turn, and random coil) from AChE were measured based on CD spectroscopic data following the online SELCON3 program (http://dichroweb.cryst.bbk.ac.uk/html/home.shtml. Accessed on: 18 August 2021) [39].

### 2.6. Cytotoxicity of Baicalein in PC12 Cells

PC12 cells were purchased from the NanJing KeyGen Biotech Co. Ltd. (Nanjing, China). These cells were cultivated in high-glucose DMEM supplemented with 10% FBS, 100 U/mL of penicillin and 100 µg/mL of streptomycin at 37 °C under a humidified 5% CO_2_ atmosphere. Baicalein was dissolved in DMSO to prepare a 5 mM stock solution and diluted with serum-free media before use. PC12 cells were seeded into a 96-well plate at the density of 2 × 10 ^4^ cells/well (100 μL) and cultured for 24 h. Next, the medium was changed, and the cells were treated with baicalein at 0, 1, 10, 20, 40, and 80 µM. Each concentration had six replicate wells, and the culture was continued for 24 h. To determine the effect of baicalein on cell viability, 20 µL of MTT (5 mg/mL) was added to each well. After 4 h, the resulting formazan crystals were dissolved by adding 150 μL of DMSO for 10–15 min. The absorption (O.D.) of each hole at 570 nm wavelength was measured using an enzyme-labelled instrument (Varioskan LUX; Thermo Fisher Scientific, Waltham, MA, USA). Cell viability was expressed as the percentage of the control.

### 2.7. Molecular Docking

Molecular docking was adopted for visualising the interaction of baicalein with AChE through the Discovery Studio 4.5 program (neotrident, Beijing, China) [40]. The crystallographic structure of the donepezil–AChE complex (PDB ID: 4EY7) was acquired through the RCSB Protein Data Bank (http://www.rcsb.org/. Accessed on: 28 September 2021) [41]. According to the special tetramer structure in AChE, Chain A was selected for simulation optimisation, and hydrogenation and polarity addition were conducted. The 3D structure of baicalein was plotted using the Chem3D Ultra 8.0 (Cambridge Soft, Waltham, MA, USA), and molecular optimisation was performed to obtain the conformation with minimum energy. Then, the CHARMm force field algorithm was used, wherein AChE and baicalein served as the receptor and ligand, respectively. Therefore, to validate the accuracy of docking, the co-crystallised ligand donepezil was re-docked into the active site of AChE. The molecular docking was performed using the CDOCKER algorithm, with 100 running times, and the docking tolerance was set to 0.25 Å. The best binding pose with the lowest energy was selected for examining the interplay of baicalein with AChE.

### 2.8. M.D. Analysis

M.D. simulation was conducted using the GROMACS 4.5.6 software, with the AMBER 99SB-ILDN force field [42,43]. The PDB file for AChE was complied with the PDB file for molecular docking. The AChE– and AChE−baicalein complex systems were solvated using the explicit SPC solvent model [44] and then placed within a dodecahedron box full of water molecules, ensuring a minimum of the 1 nm distance between all protein atoms and the box wall. The system surface charges were neutralised by adding 18 Na^+^ and nine Cl^−^, followed by energy minimisation. The minimised system was equilibrated through NVT at 300 K and one bar for 100 ps, whereas M.D. simulation was conducted by NPT ensemble. Finally, the running time of M.D. simulations was set to 60 ns [45].

### 2.9. Statistical Analysis

All samples were tested in triplicate, and the results are indicated as mean ± standard deviation. Data analysis was performed with a one-way analysis of variance in Origin 9.0 (Origin Lab, Northampton, MA, USA), and a *p*-value of <0.05 was considered statistically significant.

## 3. Results and Discussion

### 3.1. Inhibitory Effect of Baicalein on AChE

**Figure 2:**
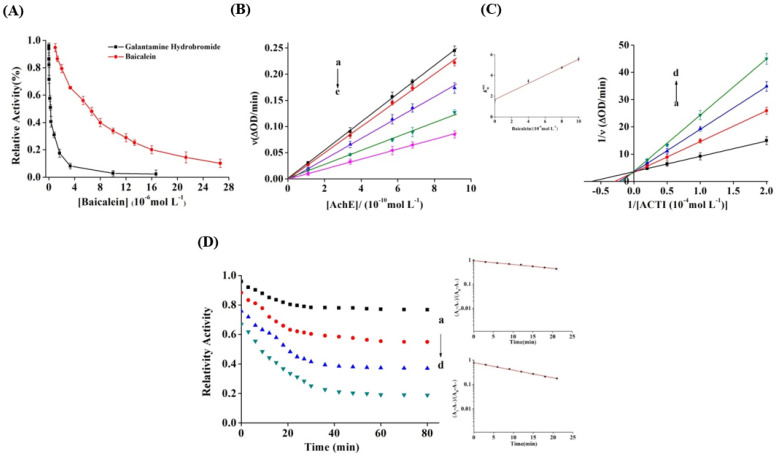
(**A**) Effect of baicalein and galantamine hydrobromide on the activity of AChE at 25 °C (pH 7.6); *c*(AChE) = 0.57 nM, *c*(ACTI) = 0.25 mM. (**B**) Plots of *v* versus (AChE). *c(*ACTI) = 0.25 mM and *c* (baicalein) = 0, 2, 4, 8, and 16 µM for curves a→e, respectively. (**C**) Lineweaver–Burk curve of baicalein, *c*(AChE) = 0.57 nM; *c* (baicalein) = 0, 4, 8, and 10 µM for curves a→d. (**D**) Time course for the relative activity of AChE in the presence of baicalein at the concentrations of 2, 4, 8, and 16 μM for curves a→d, respectively. *c*(AChE) = 0.57 nM and *c*(ACTI) = 0.25 mM. Semilogarithmic plot analysis for baicalein at 2 μM (the upper-right panel) and 16 μM (the lower-right panel), and the slope of the curves suggests the inactivation rate constants.

Baicalein and galanthamine hydrobromide (Figure 1) significantly prohibited AChE activity in a dose-dependent manner (Figure 2A), with IC_50_ values of 6.42 ± 0.07 µM and 0.29 ± 0.02 µM, respectively. The AChE activity was almost completely inhibited by galanthamine. However, AChE activity inhibition by baicalein displayed a decreasing trend. When baicalein reached a concentration of 26.7 μM, the final inhibition rate was 90%. Although baicalein inhibited AChE less effectively than galantamine, it was recognised as a compound with potential bioactivity that might prevent both Aβ aggregation and amyloid fibril plaque formation [33]. Moreover, baicalein demonstrated a lower IC_50_ value than baicalin (204.1 ± 16.5 µM), a flavone glycoside of baicalein. In vitro glycosylation of dietary flavonoids decreased plasma protein affinity [46]. Flavone glycosylation also altered the distribution and density of the electronic cloud among the rings, causing significant steric hindrance [47]. Thus, baicalein’s inhibitory activity was substantially greater than its glycosylation activity. It may be deduced that glycosylation reduces AChE inhibitory activity, and the conclusion was reached in our prior study [40]. Furthermore, the hydroxyl groups in the A ring may play a key role in AChE inhibition by baicalein, thereby supporting the docking results, suggesting that the hydroxyl groups of flavonoids form hydrogen bonds containing AChE active site residues [48].

The solvent and temperature influencing factor tests were performed to optimise the experimental conditions, considering the impact of solvent and temperature on AChE activity. No evidence of AChE inactivation was available in the ethanol concentration range of 3%, as shown in Figure S1 (supplementary data). The enzyme activity revealed a prolonged inactivation trend with an increase in the solvent concentration. Thus, the final ethanol concentration in the reaction system was regulated by 3%. The activity of AChE slowly increased between 19–37 °C and was relatively stable between 37–43 °C, whereas it decreased rapidly at >43 °C temperature (Figure S2). Baicalein is a yellow, reductive component that darkens during incubation. With an increase in the incubation temperature, the colour darkens more noticeably, interfering with the UV measurement. Therefore, the incubation temperature was adjusted to room temperature (25 °C).

### 3.2. Inhibition Kinetics of Baicalein

The plots for ν vs. AChE concentration with varying concentrations of baicalein were drawn to assess the inhibition reversibility against AChE (Figure 2B). When baicalein was added, the straight lines through the origin exhibited favourable linearity, and the slope progressively reduced, indicating that the baicalein-induced inhibition was reversible, and the binding mode was noncovalent interaction between baicalein and AChE [49]. Lineweaver–Burk plots were used to determine the inhibitory type and kinetic parameters of baicalein against AChE. The AChE activity is shown in Figure 2C at varying ATCI concentrations with varying baicalein levels. The lines of double reciprocal plots were intersected on the Y axis, which indicated a competitive inhibition, similar to chrysin’s impact on xanthine oxidase [50]. The secondary plot for $K_{m}^{\mathrm{app}}$ vs. (*I*) (insert Figure 2C) demonstrates linear fitting, assuming a single inhibition site or a single class of inhibition sites [37]. The *K_m_* and *K_i_* values obtained from Equations (1) and (2) were 1.674 and (4.32 ± 0.26) µM, respectively.

### 3.3. Inactivation Kinetics and Rate Constants

The time courses for AChE inhibition with varying concentrations of baicalein were examined in 80 min to acquire the inactivation kinetics and rate constants (Figure 2D). The figure shows that the inhibition of AChE by baicalein was time dependent. The enzyme activity reduced rapidly in the first 20 min and then gradually became steady after 40 min. Thereafter, the enzyme activity did not change dramatically. Subsequent semilogarithmic plot analysis revealed that the baicalein-induced inactivation was a monophasic first-order process with no intermediates. When the baicalein concentration ranged between 2 µM and 16 µM, the rate constants (*k*) of inactivation increased from (1.48 ± 0.03) × 10 ^−4^ s^−1^ to (5.18 ± 0.08) × 10 ^−4^ s^−1^. By using the equation ΔΔG° = −*R.T.* ln *k*, the transition free energy change (ΔΔG°) was measured as 21.85 kJ mol^−1^ s^−1^, which reduced to 18.74 kJ mol^−1^ s^−1^ upon addition of baicalein. The variation in ΔΔG° potentially induced enzyme inactivation (Table 1) [51].

### 3.4. Fluorescence Quenching of AChE by Baicalein

**Figure 3:**
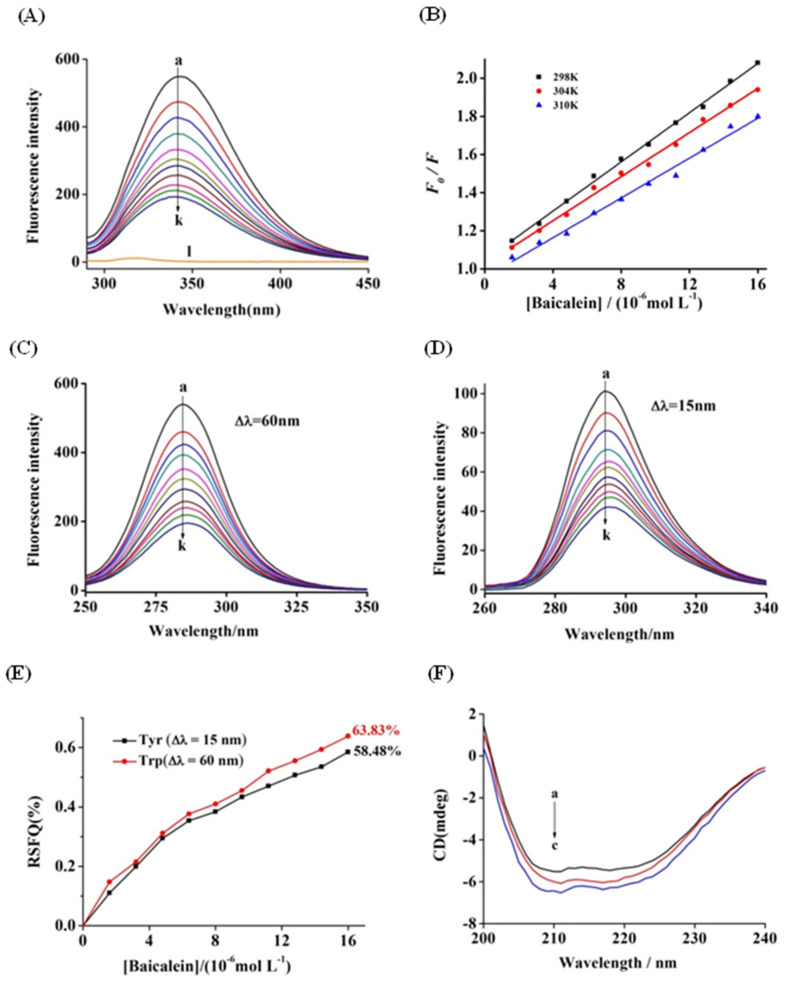
(**A**) Fluorescence spectra of AChE in the presence of baicalein at varying concentrations (pH 8.0, T = 25 °C). c(AChE) = 0.17 µM, c(baicalein) = 0, 1.6, 3.2, 4.8, 6.4, 8.0, 9.6, 11.2, 12.8, 14.4, and 16.0 μM (curves a→k), respectively. Curve l depicts the emission spectrum of baicalein alone. (**B**) The fluorescence quenching curve of baicalein on AChE at 25, 31, and 37 °C. Synchronous fluorescence of AChE when baicalein was added at different concentrations: (**C**) Δλ = 15 nm, (**D**) Δλ = 60 nm; c(AChE) = 0.17 µM, c(baicalein) = 0–16.0 μM for curves a→k. (**E**) Synchronous fluorescence quenching ratio (RSFQ = 1 − *F*/*F*_0_) of different amino acid residues of AChE. (**F**) The CD spectra of AChE in the presence of baicalein, c(AChE) = 0.75 µM; the molar ratios of baicalein to AChE were 0:1 (a), 2:1 (b), and 6:1 (c).

The remarkable inhibitory effect of baicalein on AChE indicated that the inhibitor was possibly bound to the enzyme [52]. Fluorescence quenching experiments were conducted to examine the binding property, binding constant, and binding site to further investigate the interactions between baicalein and AChE [53]. As shown in Figure 3A, the maximum fluorescence emission peak of AChE occurred at 343 nm after excitation at 280 nm, and baicalein exhibited no interference with AChE fluorescence since it did not display any signal under the same conditions (Curve l). The AChE fluorescence quenching was conducted by serially adding baicalein with no visible peak shift, implying that baicalein interacts with AChE.

The fluorescence quenching data, including quenching constant (*K*_SV_) and bimolecular quenching constant (*K_q_*), were assessed using the known Stern−Volmer equation to elucidate the quenching mechanism for AChE fluorescence through baicalein [54]. The Stern–Volmer plots exhibited favourable linearity at 25 °C, 31 °C, and 37 °C (Figure 3B), indicating the presence of a single quenching type alone. Additionally, the relevant *K*_SV_ values (shown in Table 2) reduced with an increase in temperature, proving that the fluorescence quenching mechanism through baicalein was static. Moreover, the corresponding *K_q_* values of 10^13^ L mol^−1^ s^−1^ magnitude order were substantially higher than the quenching rate constant for maximum scattering collision (2.0 × 10 ^10^ L mol^−1^ s^−1^) [55].The results also indicate that the static quenching mode triggered the quenching of baicalein to form a ground-state complex rather than a dynamic collision.

### 3.5. Binding Parameters and Thermodynamics

The binding constant (*K_a_*) for the AChE–bacalein complex and the binding site number (*n*) were measured using Equation (3) [54]:

(3) $$\log\frac{F_{0}-F}{F}=n\log K_{a}-n\log\frac{1}{\left(Q_{t}\right)-\frac{\left(F_{0}-F\right)\left(P_{t}\right)}{F_{0}}}$$

(*Q_t_*) and (*P_t_*) denote the bacalein and AChE concentrations, respectively. The order of magnitude of *K_a_* values was 10^4^ L mol^−1^ (Table 2), which indicated that bacalein bound to AChE with a moderate affinity. The *n* values approached 1, indicating the presence of merely one binding site for bacalein on AChE, which is congruent with the results of the Lineweaver−Burk plot analysis [56].

In general, four noncovalent interaction forces exist between small molecules and macromolecules, namely hydrogen bonds, van der Waals forces, electrostatic forces, and hydrophobic interactions [57]. To identify the binding forces between bacalein and AChE, the thermodynamic parameters were measured following the Van’t Hoff equation:

(4) $$\log K_{a}=-\frac{\Delta H{}^{\circ}}{2.303RT}+\frac{\Delta S{}^{\circ}}{2.303R}$$

(5) ΔG°=ΔH°−TΔS°

where *K_a_* denotes the binding constant, and *R* represents the gas constant (8.314 J mol^−1^ K^−1^). The temperatures (*T*) used were 25 °C, 31 °C, and 37 °C. As shown in Table 2, ∆*G*° and Δ*H*° showed negative values, which indicated that the interplay of bacalein with AChE was spontaneous, and the binding was exothermic. Furthermore, the positive Δ*S*° and negative Δ*H*° values indicated that the hydrogen bonds and hydrophobic interactions exerted main effects on the complex formation [58].

### 3.6. Synchronous Fluorescence Spectroscopy

For the investigation of AChE-related conformational changes, synchronous fluorescence spectroscopy is used. The fluorescence spectra between 15 and 60 nm at the wavelength interval (Δλ) indicate the fluorescence characteristics for Tyr and Trp residues and can reveal the microenvironment information of the proteases. As shown in Figure 3C,D, the increase in baicalein concentration resulted in the bathochromic shifts from 294 to 295 nm for Tyr residues and 285 to 287 nm for Trp residues of AChE. The findings indicate that the hydrophobicity of Tyr and Trp residues in the milieu was increased and that baicalein triggered a conformational change in AChE and influenced the microenvironment for amino acid residues. Tyr and Trp residues were contrasted in contribution based on their ratios in synchronous fluorescence quenching (RSFQ = 1 − *F*/*F*_0_). With an increase in the concentration of baicalein, the RSFQ value for Trp residues reached 63.83%, which was higher than that for Tyr residues (58.48%) (Figure 3E). The current finding implies that the Trp residue contributed more to the interplay between baicalein and AChE than the Tyr residue because Trp residue was closer to the binding site and had more substantial binding power with baicalein [59].

### 3.7. CD Spectra

CD may provide information on the stereochemistry of a protein-bound drug and protein conformation through secondary structure analysis, consequently revealing information about the binding process [60]. As shown in Figure 3F, two negative CD absorption bands appeared in the AChE spectra at approximately 210 and 222 nm, due to *π*→π* and *n*→π* transitions in amide groups, which have been defined as characteristic bands of the α-helix structure [55]. Moreover, the presence of baicalein increased the negative intensity of the band at 210 nm relative to the intensity of the band at 222 nm, indicating that baicalein interacted with AChE mainly by *π*→π* transition and altered the conformation of AChE. The findings of thermodynamics analysis show that the hydrophobic interactions and hydrogen bonds were the dominant forces during the interplay of baicalein with AChE. With the increase in the molar ratios of baicalein to AChE from 0:1 to 6:1, the α-helix and β-turn contents increased gradually, whereas the contents of the β-sheet and random coil decreased (Table 3). The current result is consistent with that of Manavalan et al. [61]. The observed increase in the α–helix contents indicated that the AChE structure became increasingly compact, which may have prevented the substrate from accessing the active cavity, resulting in a decrease in the AChE catalytic activity.

### 3.8. Cytotoxicity of Baicalein in PC12 Cells

**Figure 4:**
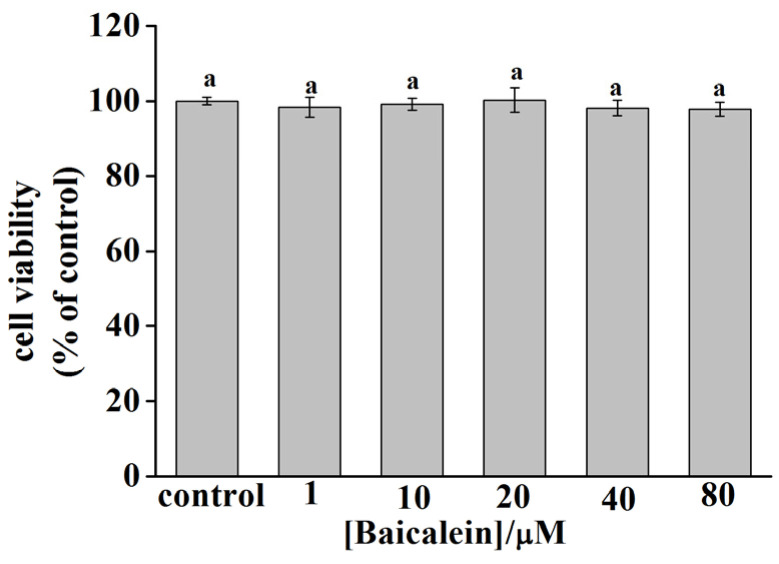
The cytotoxicity of baicalein in PC12 cells. Data are presented as the mean ± SEM of six independent experiments. The same letter “a” indicates no significant difference (*p* > 0.05).

The cytotoxicity of baicalein in PC12 cells was evaluated using the MTT assay. Figure 4 demonstrates that the viability of the PC12 cells changed slightly under different concentrations of baicalein. However, the change was not statistically significant compared with that of the control group, indicating that baicalein was nontoxic to PC12 cells at the concentration range of 1–80 µM.

### 3.9. Molecular Docking

**Figure 5:**
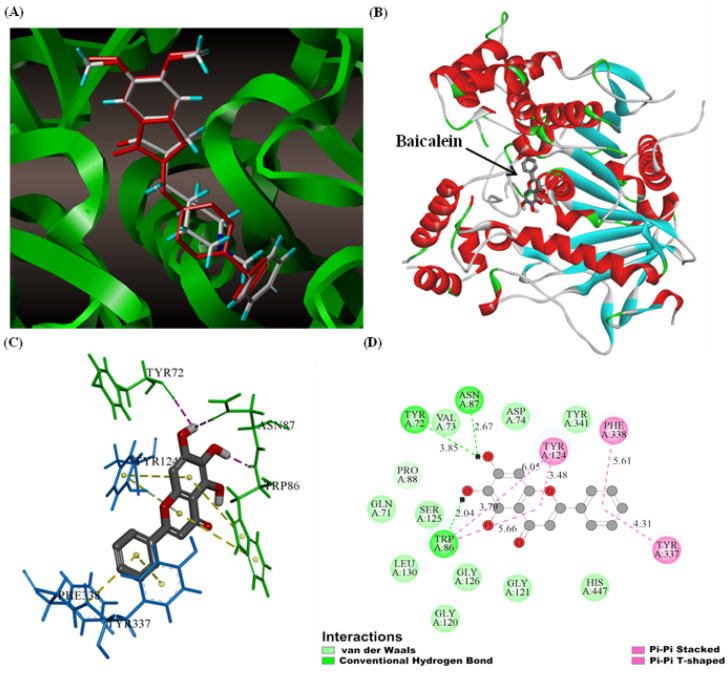
(**A**) Docked conformer of donepezil (white) and original pose of co-crystallised donepezil (red). The results of molecular docking and interaction of baicalein with AChE. (**B**) The 3D ribbon model of AChE (4EY7) docked with the optimal pose of baicalein. (**C**) The binding area of baicalein in AChE, with only the key residues shown. (**D**) The 2D schematic graphs of the main amino acid residues interacting with baicalein in AChE. The interaction is indicated in different colours.

The co-crystallised ligand donepezil was re-docked into the active site of AChE to validate the accuracy of the docking protocol. The outcome is depicted in Figure 5A, and the root mean square deviation (RMSD) between the docking and original co-crystallised pose was 0.844 Å.

The binding position, binding mode, and force type of the interaction between baicalein and AChE were predicted using a molecular simulation technique. According to the X-ray crystal structure analysis, the active site of AChE contains a narrow and deep gorge comprising two binding sites, namely the Ser-His-Glu catalytic site at the gorge bottom and peripheral anion site (PAS) at the gorge entrance.

As shown in Figure 5B, baicalein penetrated the active site of AChE, and the binding pattern was consistent with the competitive inhibition type of baicalein on AChE. Baicalein’s lowest binding energy to AChE was −33.26 kJ mol^−1^, close to the thermodynamic experimental data. Figure 5C,D demonstrate the baicalein binding area and the main amino acid residues interacting with baicalein in AChE in 3D and 2D modes. Baicalein formed three hydrogen bonds with AChE. The C6-hydroxyl group in the A ring of phenylchromen formed a hydrogen bond with the O atom of Trp86 (catalytic site) at the choline-binding site, with the bond length 2.04 Å. The C7-hydroxyl group formed two hydrogen bonds with the oxygen atom of Tyr72 and Asn87 residues, with bond lengths of 3.85 Å and 2.67 Å, respectively. The main phenylchromen had apparent *π*-*π* stacking, and hydrophobic interaction with the PAS active site residues Trp86, Tyr124, Phe338, and Tyr337 inhibited the AChE activity by suppressing aromatic inner surface induction. The Val73, Asp74, Tyr341, Pro88, Gln71, Ser125, Leu130, Gly126, Gly121, Gly120, and His447 residues surrounded the whole baicalein molecule by van der Waals forces. These residues were found in either the middle gorge area or the substrate-binding site, where they interacted with baicalein to decrease AChE activity. Furthermore, the PAS locus was linked to the allosteric control of AChE. [62]. Therefore, it is plausible to conclude that baicalein can simultaneously induce the allosteric structure of AChE, causing the gorge entrance to constrict and thereby preventing the combination of the substrate and enzyme active site and eventually inhibiting AChE activity.

### 3.10. M.D. Simulation

M.D., a typical computer simulation tool for studying protein stability, can visibly illustrate the dynamic changes of the protein skeleton with time and space. Understanding the link between enzyme structure and function is critical for understanding the inherent flexibility of proteins. As a result, M.D. simulations of the AChE crystal and the bacalein–AChE complex were run in 60 ns.

**Figure 6:**
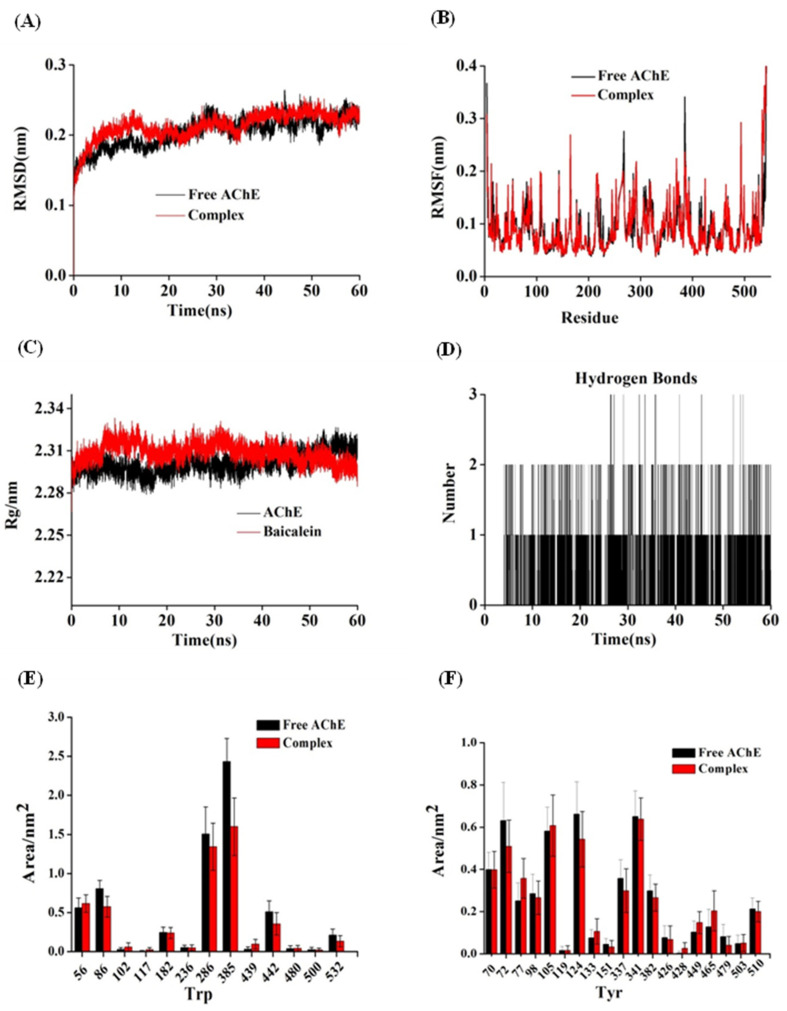
M.D. simulation of baicalein with AChE for 60 ns. The RMSD (**A**) and RMSF (**B**) plots, and Rg values (**C**) of the baicalein–AChE complex and free AChE backbone. (**D**) The number of hydrogen bonds between baicalein with AChE during simulation. The SASA values of the residues Trp (**E**) and Tyr (**F**).

The findings of the RMSD were used to evaluate the dynamic changes in system stability and determine the time when the system reaches a steady state. The RMSD values for free AChE varied from 0.15 to 0.24 nm and tended to reach equilibrium at 35 ns, as shown in Figure 6A. The RMSD value of the protein skeleton fluctuated slightly in the first 35 ns, and a stable complex was formed slowly through hydrogen bonds or intermolecular forces; the RMSD value of the enzyme became stable after 35 ns. Overall, the RMSD value of the complex did not vary greatly, indicating that the binding of baicalein and AChE would only marginally alter the freedom of protein movement and that the stability of the baicalein−AChE complex was comparable to that of AChE [63].

The flexibility of amino acid residues was demonstrated by root mean square fluctuation (RMSF) during the whole simulation duration. High RMSF values of amino acids provide high flexibility during binding. The local amino acid residues of 70−100, 110−150, and 330−380 in AChE showed an obvious fluctuation, possibly due to the participation of amino acid residues at the sites in the allosteric formation of the enzyme to form stable complexes, as is consistent with the main amino acid residues during the interaction with baicalein in molecular docking. The presence of baicalein reduced the RMSF value of AChE residues (as shown in Figure 6B), indicating that baicalein limited the flexibility of the AChE structure, which might be due to the increase in the content of the rigid structure (α-helix) of AChE due to interaction [64].

The radius of gyration (Rg) is an important parameter that reflects the compactness of protein structure [65]. After 45 ns of simulation, the Rg value tended to become stable and slightly lower than that of free AChE (Figure 6C), indicating that baicalein might induce the AChE structure to become more compact. The hydrogen bonds and other forces formed during the bonding process may have rendered the steric structure of AChE more stable, which conformed to the CD analysis results. Figure 6D depicts the variation in the number of hydrogen bonds fabricated in the complex during simulation. The hydrogen bonds varied from 0 to 3, with 1−2 being the most prevalent. The solvent-accessible surface area (SASA) of a system in interaction with solvents indicates surface changes in the system. To further verify the change in the microenvironment hydrophobicity of Trp and Tyr residues, the SASA values of the Trp and Tyr residues were compared before and after stimulation (Figure 6E,F). The SASA of Trp residue decreased noticeably, implying that Trp residues’ microenvironment hydrophobicity had improved. The overall SASA for Tyr residues, on the other hand, tended to decline slightly, which indicated a minor increase in the microenvironment hydrophobicity of Tyr residues [40]. The results corresponded with those of synchronous fluorescence research.

## 4. Conclusions

The present study investigated the inhibitory action of baicalein on AChE and the underlying mechanism at the molecular level by using several spectroscopic and computer simulation techniques. Baicalein was discovered to be a highly effective reversible competitive inhibitor of AChE. Baicalein could statically quench AChE fluorescence. The hydrogen bond and hydrophobic interaction aided the binding between baicalein and AChE. Baicalein bound to AChE at one binding site, and the order of magnitude of the binding constant was 10^4^ L mol^−1^. A cytotoxicity test showed that baicalein did not affect the activity of PC12 cells. The interaction of baicalein with PAS residues (Tyr72, Tyr124, Phe338, and Tyr337) caused an increase in the α-helix content AChE, making the structure of the enzyme more compact and increasing the surface hydrophobicity of AChE. Through hydrogen bonding, hydrophobic interaction, and van der Waals force, baicalein may embed into the active site of AChE to form a relatively stable complex. The binding caused a conformational shift in the enzyme and tightened the structure of the gorge entrance, preventing the substrate from entering and binding with the enzyme’s active site, eventually inhibiting AChE activity. M.D. simulation analysis showed that the binding of baicalein with AChE might influence the stability of the protein skeleton. As fluctuating peptides, the 70–100, 110–150, and 330–380 residues might participate in causing conformational changes in the enzyme and forming stable complexes. Furthermore, a slight decrease in the Rg value reflects a moderate compact structure of AChE. Overall, the current study investigated the mechanism of AChE inhibition by baicalein and provided a new direction for the research and development of anti-AD food functional factors and drugs.

## Figures and Tables

**Table 1:** Inactivation rate constants for AChE with baicalein.

Baicalein (µM)	Inactivation Rate Constant *^a^* (×10^−4^ s^−1^)	Transition Free Energy Change *^b^* (kJ mol^−1^ s^−1^)
	*k*	
2	1.48 ± 0.03	21.85
4	2.42 ± 0.05	20.63
8	4.01 ± 0.02	19.38
16	5.18 ± 0.08	18.74

*^a^k* is the first-order rate constant. The values of *k* were significantly different (*p* < 0.05) from each other; *^b^* Transition free-energy change, ΔΔ*G*° = −*R.T.* ln*k*, where *k* is the time constant of the inactivation reaction.

**Table 2:** The quenching constants (*K*_SV_), binding constants (*K_a_*), and relative thermodynamic parameters for baicalein−AChE interaction under three temperature conditions.

*T* (°C)	*K*_SV_ (×10^4^ L mol^−1^)	*R* ^a^	*K_a_* (×10^4^ L mol^−1^)	*R* ^b^	*n*	∆*H*° (kJ mol^−1^)	∆*G*° (kJ mol^−1^)	∆*S*° (J mol^−1^ K^−1^)
25	6.45 ± 0.02	0.9989	6.66 ± 0.05	0.9982	0.88 ± 0.01	−17.01 ± 0.02	−22.71 ± 0.06	19.15 ± 0.09
31	5.78± 0.03	0.9972	5.84 ± 0.01	0.9979	0.93 ± 0.03		−22.83 ± 0.03	
37	5.22± 0.07	0.9946	5.11 ± 0.04	0.9967	1.13 ± 0.02		−22.94 ± 0.08	

*R*^a^ indicates the correlation coefficient of the *K*_SV_ values; *R* ^b^ represents the correlation coefficient of the *K_a_* values.

**Table 3:** Secondary structure contents of the baicalein–AChE complex of varying molar ratios.

Molar Ratio (Baicalein):(AChE)	α-Helix (%)	β-Sheet (%)	β-Turn (%)	Random Coil (%)
0:1	34.35 ± 0.74	18.62 ± 0.06	19.18 ± 0.28	28.02 ± 0.65
2:1	38.82 ± 0.08	14.57 ± 0.13	21.42 ± 0.57	25.36 ± 0.29
6:1	42.53 ± 0.35	7.73 ± 0.03	28.15 ± 0.89	21.71 ± 0.47

## Supplementary Materials

The following supporting information can be downloaded at: https://www.mdpi.com/article/10.3390/foods11020168/s1, Figure S1: Effect of ethanol on AChE activity; Figure S2: Effect of temperature on AChE activity.
