# Supplementary material for: Inhibitory Mechanism of Baicalein on Acetylcholinesterase: Inhibitory Interaction, Conformational Change, and Computational Simulation
Source: Foods. 2022 Jan 10;11(2):168. doi: 10.3390/foods11020168 (PMC8774682; doi:10.3390/foods11020168)
Supplement: Supplementary file 1 [file foods-11-00168-s001.zip › foods-1512897-SI.pdf]

Supplementary data

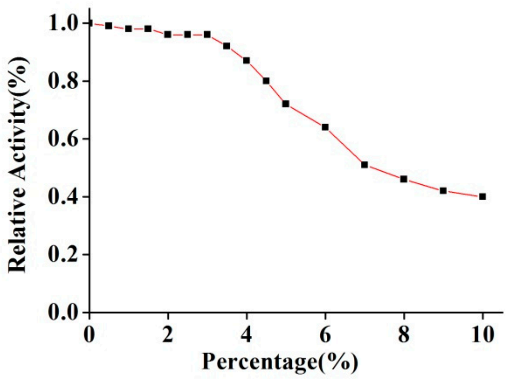

Figure S1. Effect of ethanol on AChE activity.

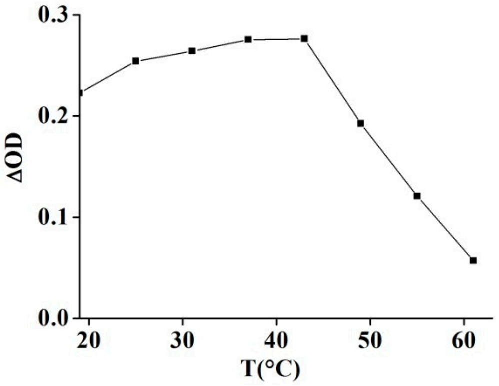

Figure S2. Effect of temperature on AChE activity.
